# Fault Diagnosis Method for Axial Piston Pump Slipper Wear Based on Symmetric Dot Pattern and Multi-Channel Densely Connected Convolutional Networks

**DOI:** 10.3390/s25247465

**Published:** 2025-12-08

**Authors:** Huijiang An, Honghan He, Shihao Ma, Ruoxin Pan, Cunbo Liu, Yuxuan Guo, Gang Liu, Mingxing Song, Zhikui Dong, Gexin Chen

**Affiliations:** 1 School of Mechanical Engineering, Yanshan University, Qinhuangdao 066004, China; 2 Silesian College of Intelligent Science and Engineering, Yanshan University, Qinhuangdao 066004, China; 3 School of Mechanical Engineering, Hebei University of Architecture, Zhangjiakou 075000, China; 4 Mechanical and Electrical Engineering, Xinjiang Institute of Engineering, Urumqi 830023, China

**Keywords:** axial piston pump, slipper wear, fault diagnosis, symmetrized dot pattern, densely connected convolutional networks

## Abstract

Fault diagnosis in axial piston pumps is key to ensuring the proper operation of a hydraulic system. Slipper wear, as a typical fault in piston pumps, is challenging to accurately diagnose because the faults are very similar for different forms and degrees of wear. The achievement of accurate fault diagnosis of different forms and degrees of wear in the slipper will greatly improve the reliability of axial piston pump operation and, at the same time, provide new ideas for research into similar fault diagnosis problems in other rotating machinery. Therefore, a method of fault diagnosis based on the following symmetric dot pattern (SDP) and multi-channel densely connected convolutional networks (DenseNet) is proposed in this paper. The method applies an SDP transformation to transform the slipper failure signal into an SDP image, which achieves the fusion of triaxial vibration signals and enriches the signal features. The inception module is improved by replacing the original structure with larger convolutional kernels in multiple branches and decomposing the larger convolutional kernels. The inception module, the convolutional block attention module (CBAM), and the DropBlock method are introduced into DenseNet to improve feature extraction capability, computational efficiency, and model generalization ability. Experiments are performed on several slipper wear fault SDP image datasets, and all the indices produced by the proposed method are higher than those of the traditional convolutional neural networks, which fully proves the effectiveness and superiority of the procedure.

## 1. Introduction

Axial piston pumps are generally used in aerospace hydraulic systems, construction machinery, and ocean engineering. The slipper is an important part of the piston pump. It is subjected to a number of unstable forces from the piston and the swash plate, making it the most vulnerable component to wear and failure in the piston pump [1,2,3,4]. Slipper wear can not only shorten the service life of the piston pump but also lead to its catastrophic failure [5]. Therefore, the study of fault diagnosis algorithms for multiple forms of wear and different degrees of wear on the slipper is of great value in promoting the improvement and development of piston pump maintenance technology [6].

**Figure 1:**
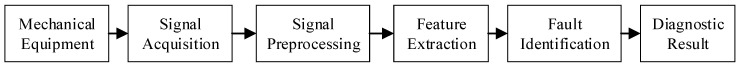
Intelligent fault diagnosis process.

In recent years, deep learning has generally been used in piston pump fault diagnosis for its powerful ability to mine and extract useful features [7]. Figure 1 shows the intelligent fault diagnosis process [8,9]. Commonly used models, such as convolutional neural networks [10], residual networks [11], and Transformer [12], are constantly being improved and fused and then applied to fault diagnosis tasks [13,14,15]. He et al. [16] proposed an adversarial model-based transfer learning system for deep multi-signal fusion with good performance for cross-domain piston pump fault diagnosis. Tang et al. [17] integrated the theory of deep learning and the Bayesian optimization algorithm to accurately distinguish the different fault conditions of piston pumps. Ugli et al. [18] proposed an automatic optimization approach for the fault diagnosis of piston pumps using a one-dimensional convolutional neural network (1D-CNN) architecture and a genetic algorithm through the inclusion of direct links and a dimension reduction model within the 1D-CNN block, which performed well on various kinds of fault diagnosis tasks, for example, slipper wear and thrust plate wear.

Guo et al. [19] proposed a hybrid model of deep learning and WaveletKernelNet-CBAM-BiLSTM for troubleshooting drilling pumps. The method utilizes random forests for signal selection, integrates CBAM to enhance feature representation, and employs BiLSTM for temporal data processing. Bayesian optimization is applied to fine-tune the hyperparameters. The experimental results demonstrate the accuracy of the model, as well as its reliability and generalization capability. However, this method diagnoses faults that are caused by varying numbers of worn slippers and that exhibit significant dissimilarities; it does not account for similar wear faults in a single slipper. Hatami Garousi et al. [20] proposed a vibration-based method of diagnosing centrifugal pump failures, utilizing a multi-layer perceptron (MLP) to classify faults. The study extracts time- and frequency-domain features from vibration signals, demonstrating that statistical indicators like kurtosis and skewness effectively distinguish defects such as impeller damage and cavitation. Experimental results validate the method’s ability to detect and quantify fault severity in real time. Du et al. [21] proposed a novel approach to rotor fault diagnosis and isolation based on search coils for permanent magnet synchronous motors, in which two random forest models are employed to achieve automated classification for distinguishing between two faults with similar characteristics. However, the method only considered two fault forms and depended on sensor placement and signal processing. Wang et al. [22] proposed a deep learning way based on transfer adversarial subnetworks and channel-wise thresholds. However, the method is effective in classifying two kinds of faults, namely, gearwheel tooth wear and pinion missing teeth, and it is difficult to prove its effectiveness in discriminating similar features. Ding et al. [23] proposed a classifier prediction-oriented domain adaptation network that aims to solve the decision boundary confusion caused by strong connectivity among similar categories. However, the method focused on solving the cross-domain fault diagnosis problem and did not directly solve the similar fault diagnosis problem. Table 1 summarizes the literature review.

**Figure 2:**
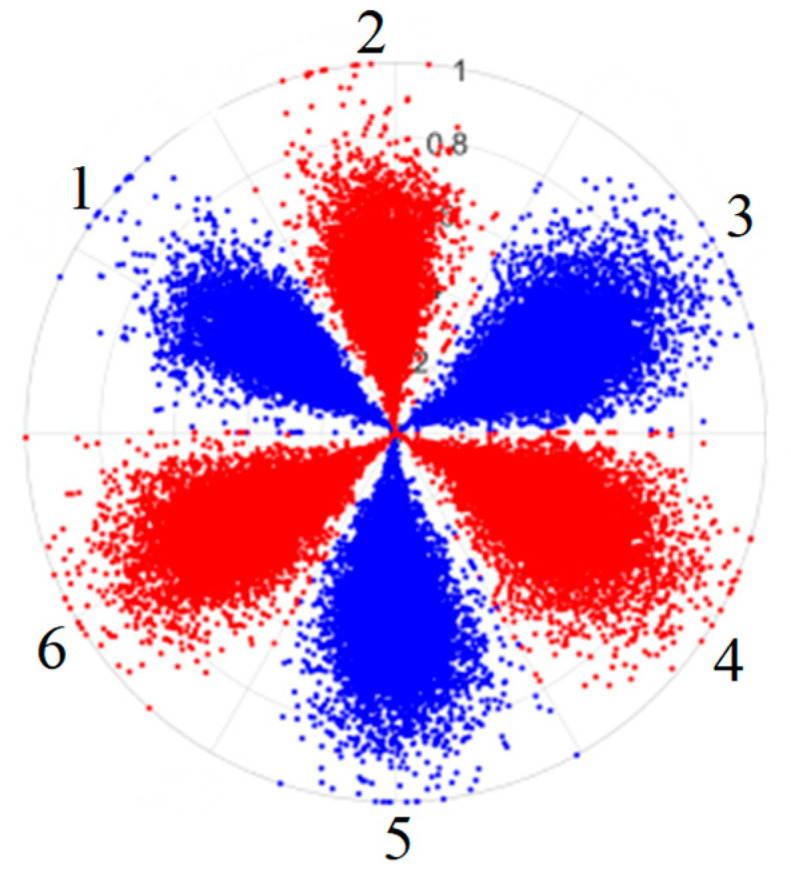
Example of triaxial fusion via SDP.

To address these challenges, this study introduces SDP transformation as a superior alternative to conventional time–frequency representations like spectrograms or wavelet transforms. While spectrograms and wavelets are powerful, they often struggle with low signal-to-noise ratios and generate high-dimensional, complex outputs that can be computationally intensive for deep learning models to process, especially when fusing multiple sensor channels. In contrast, the SDP technique offers distinct advantages: (1) it inherently possesses strong noise robustness, effectively suppressing stochastic noise in weak fault signals; (2) most critically for this application, SDP provides a novel and intuitive framework for fusing triaxial vibration signals into a single, unified two-dimensional image. This fusion capability uniquely preserves the synergistic relationships and composite fault patterns across different axes, which are often critical for diagnosing complex faults like the multiple wear forms in piston pump slippers. A visual example of this triaxial fusion via SDP is presented in Figure 2. In the SDP image, each vibration signal is transformed into symmetrical “petals”—specifically, the triaxial vibration signals are mapped to three pairs of symmetrical petals (e.g., petals 1 and 2 correspond to the X axis). Each pair is distinguished by red and blue colors to facilitate the analysis and comparison of features from the same axis.

In summary, signal processing and analysis methods such as wavelet packet transform, cyclostationary theory, and methods such as deep learning and domain adaptive algorithms are helpful in solving similar fault diagnosis problems. However, due to the complex structure of axial piston pumps, more forms of slipper wear, different wear degrees, and unclear wear mechanisms tend to occur, and thus, the fault diagnosis research for slipper wear and similar faults is still less. Multiple slipper wear forms, different wear degrees, and different composite wear forms mean that the signals produced by the system are weak; noise and data change trends are similar; feature information performance is similar; feature extraction is difficult; and thus, the existing fault diagnosis models struggle to diagnose these slipper wear faults and other similar faults. Therefore, the existing fault diagnosis methods need to be improved in terms of signal processing and network structure. In this paper, we will focus on three different wear forms that occur in axial piston pump slippers—abrasive wear, corrosive wear, and adhesive wear—as well as three diverse degrees of wear failure: slight, moderate, and serious.

To this end, a fault diagnosis method based on SDP with multichannel DenseNet is proposed in this article. The method effectively enhances the feature extraction capacity of the network and the generalization capacity of the model, performs well with regard to the fault diagnosis of different forms and degrees of wear in the slipper, and provides new ideas for solving other similar fault diagnosis problems. The contributions of this study can be summarized as follows:

(1) The SDP image transformation method is applied to the construction of image samples of a slipper fault for the first time, and the triaxial vibration signal is directly transformed into an SDP image, which achieves the feature fusion of triaxial vibration signals, enriches the features of the fault diagnosis sample, and reduces the time complexity.
(2) Based on the design of the inception module, conventional fault diagnosis methods are improved by replacing their original structure with a larger convolutional kernel and multi-branching that decomposes the larger convolutional kernel, which enriches feature information under different sensory fields and effectively captures both local and global features.
(3) The improved inception module, CBAM, and DropBlock methods are introduced into DenseNet, and DenseBlock4 in the original DenseNet is removed to establish a multichannel DenseNet-based fault diagnosis model. The model has a high identification accuracy in fault diagnosis tasks for multiple wear forms, different wear degrees, and different composite wear forms of the slipper.

The remainder of the article is scheduled as follows: Section 2 presents the related theory. Section 3 describes the structure of the proposed method. In Section 4, the experimental details and results analysis are presented. Finally, Section 5 concludes the paper.

## 2. Preliminaries

### 2.1. Symmetric Dot Pattern Transformation (SDP)

SDP is an image representation approach based on the polar coordinate system, which can directly transform an original signal into an SDP image. The method is simple to operate, low in computational requirements, and robust against noise, and it is able to clearly show the amplitude, phase, and frequency of the vibration signal and other key information so that the fault characteristics can be expressed intuitively [24,25,26,27]. At the same time, the method can also achieve multi-channel data fusion, which can be used in the fault diagnosis collar to reduce the workload of the algorithm to recognize the type, location, and extent of the fault.

**Figure 3:**
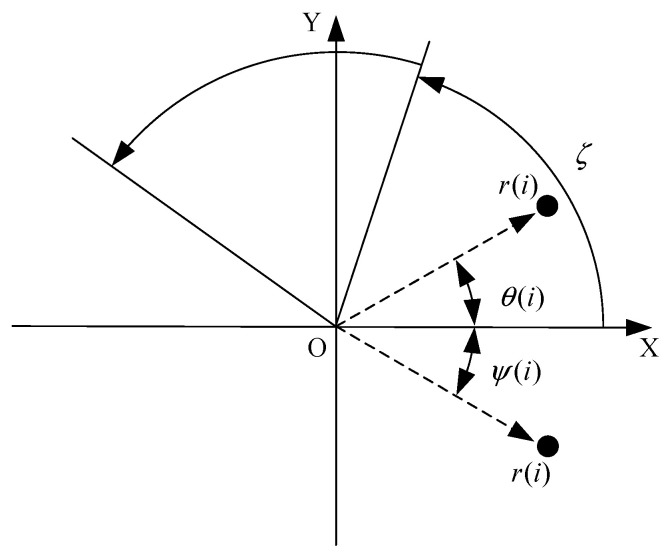
Principle diagram of SDP method.

For the obtained time domain signal $X=\left\{x_{1},x_{2},\ldots ,x_{n-1},x_{n}\right\}$, $x_{i}$ denotes the signal amplitude at the moment of i, $x_{i+l}$ denotes the signal amplitude at the moment of i+l, and the time domain signal can be transformed into polar coordinates through normalization to generate the corresponding SDP image. Figure 3 shows the basic principle of the SDP image conversion method, r(i) is the polar radius, θ(i) is the angle of counterclockwise rotation of the polar coordinates along the line, and ϕ(i) is the angle of clockwise rotation of the polar coordinates along the initial line. The relation between the amplitude and frequency of the time domain signal can be simply and directly mapped to the polar coordinate space through Formulas (1)–(3), and fault information included in the time domain signal can be presented through the resulting image. The mapping relationship is given by the following:

(1) $$r(i)=\frac{x_{i}-x_{\max}}{x_{\max}-x_{\min}}$$

(2) $$\theta (i)=\theta +\frac{x_{i+l}-x_{\min}}{x_{\max}-x_{\min}}\zeta$$

(3) $$\psi (i)=\theta -\frac{x_{i+l}-x_{\min}}{x_{\max}-x_{\min}}\zeta$$

where $x_{i}$ is the ith sample amplitude of the signal, $x_{\min}$ is the minimum amplitude of the signal, $x_{\max}$ is the maximum amplitude of the signal, l is the time interval parameter, θ is the rotation angle of the mirror plane of symmetry, θ=360°/m,m=1,2,…,n, ξ is the angular amplification factor.

To provide a clearer algorithmic description of the SDP transformation, particularly for triaxial vibration signals (X, Y, Z axes), the procedure is summarized as follows. First, the individual time-domain signals from each axis are normalized to a uniform scale. Subsequently, these normalized signals are mapped into the polar coordinate system using Equations (1)–(3). Specifically, for each data point in the signal, a corresponding point is plotted in the polar plane, with its radius determined by the signal amplitude and its angle by the time index and the predefined rotation parameters. Finally, by plotting all the transformed points from all three axes onto a single polar plane and connecting them with smooth lines or distinct markers, a composite SDP image is generated. This image effectively fuses the multi-channel vibration information, creating a unique pattern that visually represents the underlying fault characteristics.

As shown in Figure 3, the coordinate axis OX is the polar axis and r(i) is the polar radius, and the signal amplitude at a certain point of the signal can be expressed at the position shown in the figure by combining the angular amplification factor ξ. θ(i) and ψ(i) are the two deflection angles about the mirror plane of symmetry, and their deflection angles are symmetrical about the polar axis.

### 2.2. DenseNet

The main idea of DenseNet is to introduce dense connections into the network, which can effectively achieve and strengthen feature reuse and enhance feature propagation between the front and back layers of the network. This allows the network to make better use of the shallow feature information so as to raise the network’s performance and efficiently prevent the model from encountering degradation and overfitting problems [28,29,30].

**Figure 4:**
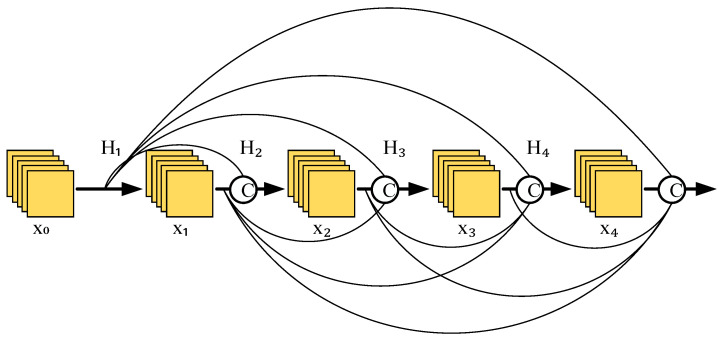
Dense connection structure.

In a traditional convolutional neural network, if the network has L layers, it will produce L connections, i.e., the inputs of each layer come from the outputs of the previous layer of the network. However, the dense connections introduced in DenseNet allow L layers of the network to produce L(L+1)/2 connections, i.e., the inputs of each layer receive the outputs of all the first few layers, allowing the features extracted from each layer to be effectively used. Figure 4 represents the basic structure of a dense connection.

From Figure 4, $x_{0}$ is the initial data input, and the input of $H_{1}$ is $x_{0}$; the inputs of $H_{2}$ are $x_{0}$ and $x_{1}$; and the inputs of layer i can be obtained by the following analogy: $x_{i}=H_{i}\left(\left[x_{0},x_{1},\ldots ,x_{i-1}\right]\right)$. Where $H\left(\cdot \right)$ indicates a nonlinear transformation function, it is a combination of operations that might involve an array of batch normalization, activation, pooling, and convolution manipulations, and it should be noted that there may actually be more than one convolutional layer between layers i and i−1 here.

**Figure 5:**
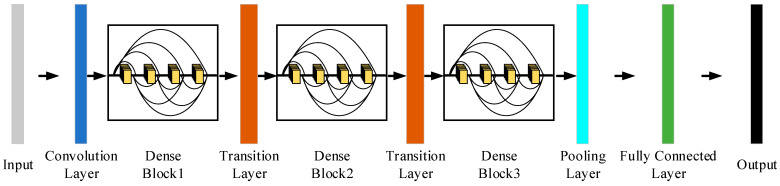
DenseNet structure.

Convolutional neural networks generally go through a pooling layer or a convolution operation with a step size greater than 1 to reduce the size of the characteristic diagram, whereas DenseNet’s special dense connection approach requires that the size of the characteristic diagram remain consistent. To overcome this challenge, the structure of the alternate connection of the dense block and transition layers is used in DenseNet, where the dense block is a module containing many layers, each layer has the same size as the characteristic diagram, and the layers are densely connected to each other. The role of the transition layer is to link two neighboring dense blocks and to reduce the characteristic diagram size by pooling operations. Figure 5 shows the DenseNet structure.

### 2.3. Inception Module

**Figure 6:**
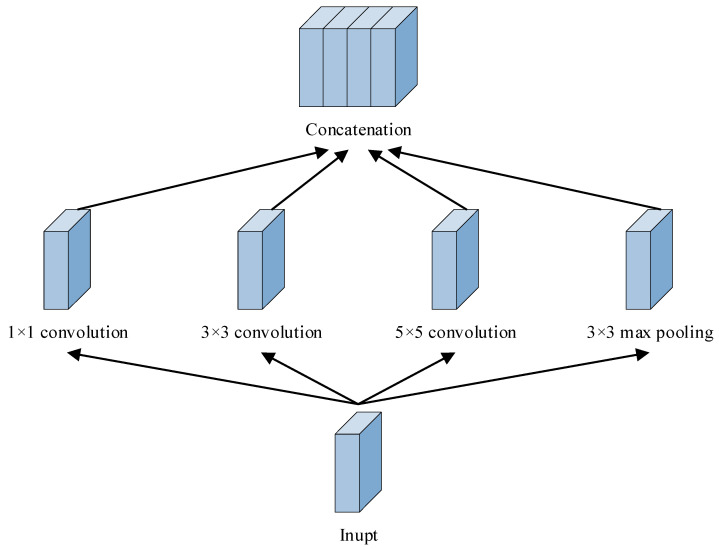
Inception module structure.

The inception module is the most central network substructure in GoogLeNet, with a structure designed to capture features at different scales and levels, being able to retain more features in the input signal. The design of the inception module is based on the following core idea: a parallel extraction effort of the input signal using multiple convolutional kernels of diverse sizes and pooling layers, and then stacking the results of this multi-branch convolution and passing them on to subsequent input layers. It uses different-sized convolutional kernels in parallel within a layer, as well as maximum pooling operations, to be able to capture features at different scales of the image without significantly increasing the computational burden. Figure 6 shows the Inception module structure [31,32].

## 3. Proposed Method

### 3.1. Improved Inception Module

**Figure 7:**
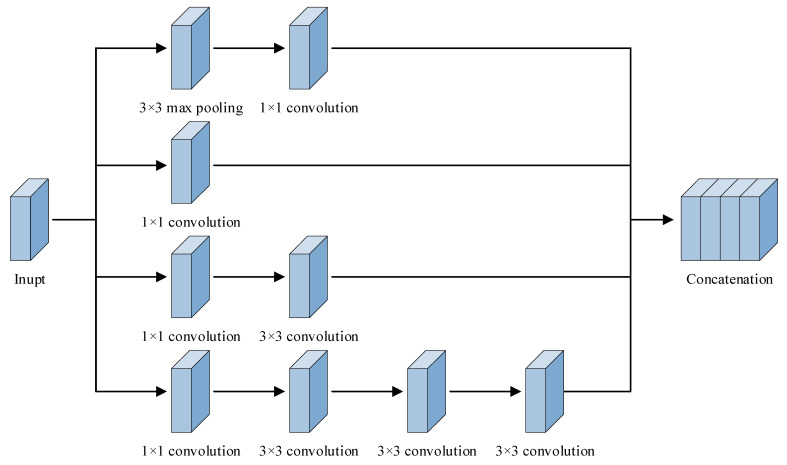
Improved inception module structure.

To raise the feature extraction capacity of the neural network, this article is based on the design idea of the inception module and aims to improve the existing inception module. The idea for improvement is to replace the original structure with a larger convolutional kernel in a multi-branching format and then decompose the larger convolution kernel in order to decrease the parameter calculations. Figure 7 shows the improved inception structure.

The overall structure of the improvement module is as follows: three sizes of convolutional kernels, 1 × 1, 3 × 3, and 7 × 7, are designed to perform multi-scale parallel feature extraction on the input image so that it can retain feature information at multiple scales. The improved module is analyzed line by line from bottom to top: the first line is a combination of the 1 × 1 convolution and 7 × 7 convolution, which uses a larger sensory field to convolve the original signal and retains the large-scale information, and to decrease parameter calculations for the large-scale convolution, the 7 × 7 convolution is decomposed into a combination of three 3 × 3 convolutions without changing the size of the characteristic diagram. The second line is a combination of the 1 × 1 convolution and 3 × 3 convolution using a medium-scale convolutional kernel to extract features on a small scale for the input features. The third line, 1 × 1 convolution, uses a small-scale convolutional kernel for performing lightweight feature extraction and dimension reduction operations. The fourth line is a combination of 3 × 3 pooling and 1 × 1 convolution, which helps in extracting local information from the input signal.

The improved inception module is able to perform multi-scale feature extraction on the input signal, and the feature outputs of the four branches are spliced in the channel dimension, which maximally enriches the feature information under different sensory fields without changing the height and width of the feature matrix. The decomposition operation of the large convolutional kernel greatly reduces the amount of parameter computation during the convolutional process, which provides good feature data for the subsequent neural network for fault diagnosis.

### 3.2. DenseNet-I

In this article, we use the DenseNet architecture to build a fault diagnosis model for slipper wear fault diagnosis. Firstly, the improved inception module is inserted into the forefront of DenseNet to strengthen the feature extraction capacity of the original signal, maximize the feature information of the original signal to be retained, and form the output with rich feature representation, which enhances the robustness of the model against diverse scales of information to some degree and, in the meantime, improves its computational efficiency.

The DropBlock approach [33] is then added to the convolutional layers in each dense block of DenseNet as a regularization method to optimize the overall network. The basic mechanism is to deactivate a space block, which is a part of the characteristic diagram, by selecting it and setting the characteristic values in that block to zero. It is more targeted at the spatial level, and can remove a block of information and deactivate spatial blocks with greater probability, which introduces additional regularization effects and helps the network generalize better to new data. The formula for the DropBlock method is as follows:

(4) $$\gamma =\frac{(1-keep\_prob)\times {\left(feat\_size\right)}^{2}}{{\left(block\_size\right)}^{2}\times (feat\_size-block\_size+1)}$$

where *γ* is the value of the Bernoulli function probability, i.e., the probability of the discard process, which affects the strength of the regularization, keep_prob is the probability of each block feature being retained, feat_size is the size of the feature map, and block_size is the length and width of the neuron squares that were discarded.

This approach not only helps the network to learn and exploit features better but also reduces the risk of overfitting and improves the generalization capability of the network. In the meantime, the CBAM is added between the dense block and the transition layer in DenseNet. CBAM is made up of a channel attention module and a spatial attention module. The feature vectors extracted in the previous level through the convolutional layer are weighted by CBAM, which performs weighting operations on them in both the channel and spatial dimensions to enable adaptive feature selection and enhancement. It highlights the main features and suppresses extraneous features so that the model pays more attention to the content and positional message of the target that needs to be detected, so as to raise its diagnostic accuracy. ReLU is used as the activation function in both the dense block and transition layer in traditional DenseNet. Considering its issues, for example, easily generating gradient disappearance and neuronal node death, this study employs the Leaky ReLU function [34] as the activation function throughout DenseNet.

**Figure 8:**
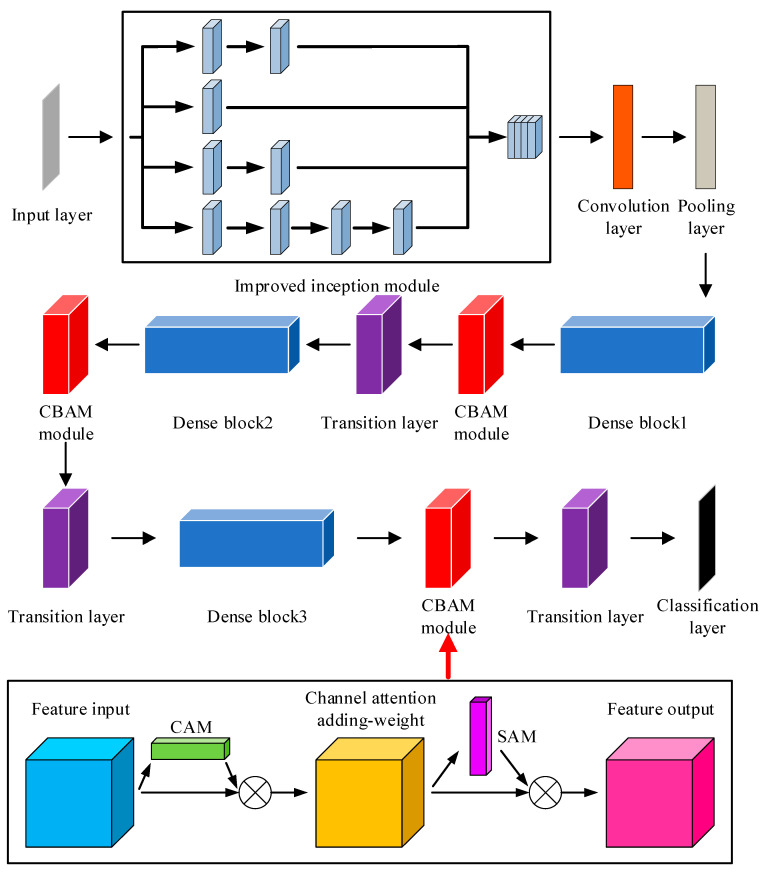
The structure of DenseNet-I.

Considering that DenseNet contains a total of four dense blocks, which can classify 1000 kinds of images, this paper only needs to achieve the goal of fault diagnosis for four different forms of slipper wear to reduce the number of neural network layers and speed up the calculation, accelerating the model. After adding a variety of modules to improve the original DenseNet, DenseBlock4 is deleted, and the improved network is named DenseNet-I. On the one hand, the dense connection of DenseNet enhances the feature propagation between layers and promotes the extraction of rich feature messages. On the other hand, the features extracted by the network are able to perform channel and space weighting operations for the desired features after passing through two layers of the CBAM, which enormously enhances the expressive power of the network. Table 2 shows the overall structure and parameters of DenseNet-I. Figure 8 shows the network structure.

**Figure 9:**
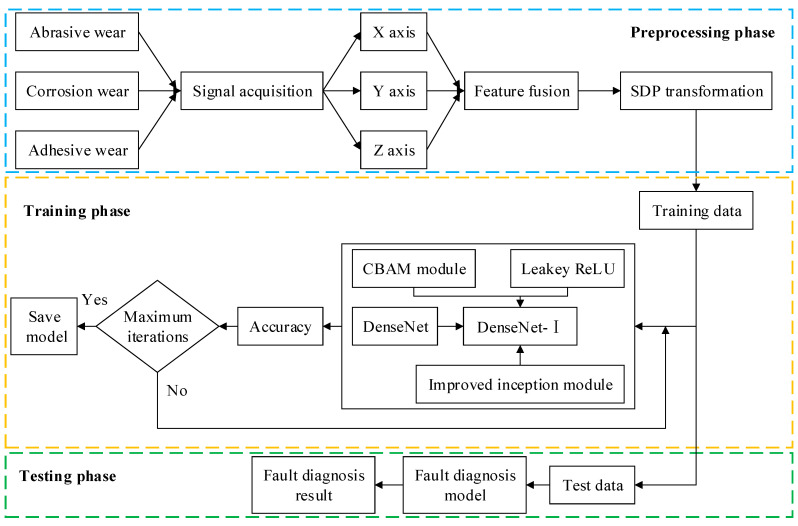
Fault diagnosis process.

Figure 9 shows the fault diagnosis process. Firstly, the triaxial vibration signals are converted into SDP images and constitute the dataset. The dataset is subdivided into a training set and a test set. Then, DenseNet-I is trained on the training set to achieve parameter optimality. Finally, the trained model is tested on the test set. Compared with the fault diagnosis method applied in traditional convolutional neural networks, the proposed method derives richer information from the input samples, has a stronger feature extraction capacity, achieves more efficient feature transfer, slows down the gradient vanishing problem, and promotes feature reuse.

## 4. Experimental Results and Analysis

### 4.1. Experiment Description

**Figure 10:**
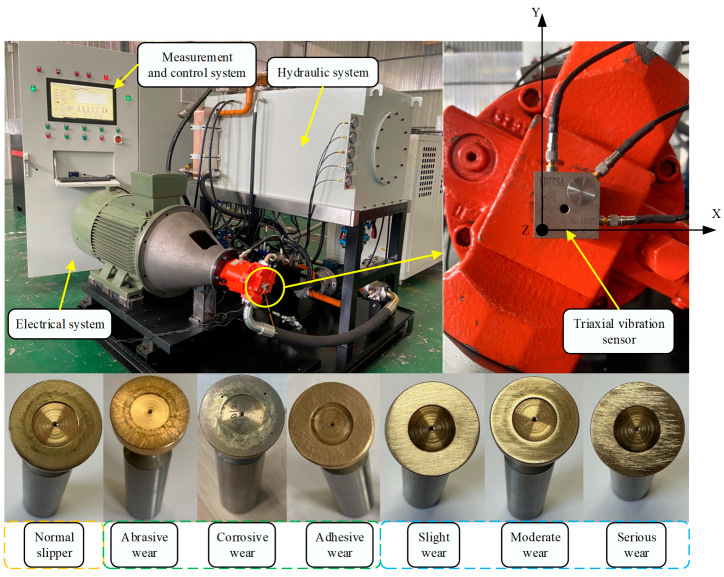
Test bench and faulty parts.

This experiment uses an A10V71DRG/31L (Bosch Rexroth AG, Stuttgart, Germany) swashplate axial piston pump, according to its performance parameters, structural characteristics, and failure simulation experimental needs, to build the piston pump slipper fault simulation test bench shown in Figure 10. The test bench can be subdivided into three parts by its functional composition: a hydraulic system, a measurement and control system, and an electronic control system. The control voltage of the inverter can be altered in real time on the user interface of the test bench, thus changing the speed of the piston pump according to the experimental requirements. The control current of the relief valve can also be changed to achieve different pressure requirements of the system. Meanwhile, the rational allocation of the data acquisition board can achieve the acquisition and storage of key signals such as the pressure, flow, and vibration of the system. The faulty parts are prepared based on the mechanisms of different forms of slipper wear and the changes in the friction coefficient during the wear process of the slipper pair. Figure 10 shows the slipper’s faulty parts with different wear forms and diverse degrees of wear.

Slider wear failure signals are captured by using the YD35D triaxial vibration sensor. The triaxial vibration sensor is installed with a magnetic base in the center of the piston pump end cover so that the sensor is longitudinally parallel to the ground to ensure that its X, Y, and Z axes are correctly positioned in space; the vibration sensor arrangement is shown in Figure 10.

In order to collect more information about the piston pump slipper wear faults, along with the parameters of the data acquisition board, the acquisition frequency of the triaxial vibration signal was set to 20 kHz.

The acquisition process was chosen to be carried out at a pressure of 28 MPa, the rotational speed was set to 1500 r/min, and the sampling time was set to 60 s.

### 4.2. SDP Image Dataset Construction

**Figure 11:**
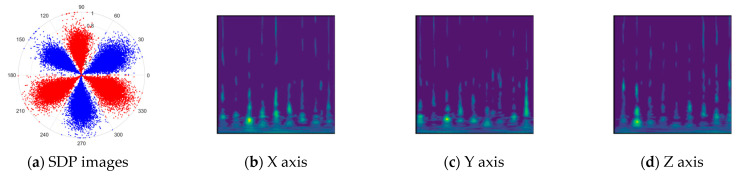
Comparison between SDP images and CWT transformed images.

Figure 11 shows a comparison between the Continuous Wavelet Transform (CWT) and the SDP transformation. The SDP method generates a single composite image by fusing the triaxial signals, whereas CWT produces three separate time–frequency representations. The SDP image offers a more compact and computationally efficient representation, consolidating fault features from all three channels into a single input for the neural network, thereby simplifying the network architecture and improving diagnostic efficiency.

Table 3 shows the results of a controlled variable experiment that systematically analyzes the influence of various parameters on the SDP petal morphology, conducted with an empirical value of θ=60°. Based on these findings, the final conversion parameters were determined to be θ=60°, l=7, and ξ=50°.

The collected triaxial vibration signals of four different wear forms of the slipper are used to construct the dataset, taking the signals under the normal state of the slipper as an example, and the dataset is constructed according to the following steps:

(1) The triaxial vibration signals acquired at an acquisition frequency of 20 kHz over a period of 60 s are arranged in the order of X, Y, and Z to form a two-dimensional array of samples in three columns;
(2) With 1024 as the data length for the two-dimensional array of three columns of data, at the same time, for successive extraction in accordance with θ=60°, l=7 and ξ=50° are the key parameters of the SDP image conversion of the three columns of data fusion, constituting a fault sample image [27];
(3) A total of 1000 sample images are constructed by performing 1000 extractions of a two-dimensional array and performing SDP image conversion according to the above parameters;
(4) The above 1000 samples are subdivided into training and test sets of the model in the ratio of 9:1.

The remaining slipper wear fault data follow the above steps, and Table 4 shows the deep learning dataset of the piston pump slipper faults.

**Figure 12:**
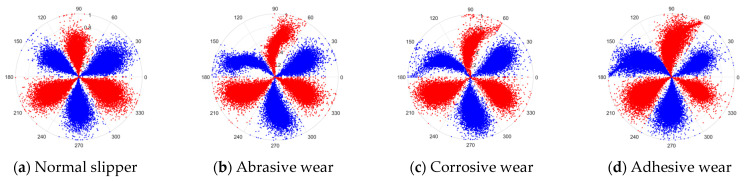
SDP images of vibration signals of different wear forms of the slipper (the red and blue colors distinguish petals from the same axis for comparative analysis).

There are 1000 samples for each of the four different slipper wear states: normal slipper, abrasive wear, corrosive wear, and adhesive wear. After fusion, each SDP sample image contains 1024 sample points in each of the three axes of X, Y, and Z. There are a total of 1000 sample images for each type of fault. The results of the SDP fusion of the vibration signals of a randomly selected set of slipper wear cases in different wear forms are shown in Figure 12.

### 4.3. Modules Validity Experiments

The model was trained for 100 epochs with drop_size=0.1, block_size=7, a batch size of 64, and a learning rate of 0.01.

To study the influence of each module in the proposed DenseNet-I on the effectiveness of the overall model’s fault diagnosis, the improved Inception module, the CBAM, and the DropBlock method are used as an ordered combination of 3 modules for the comparison experiments, and 10 experiments are averaged. Table 5 shows the experimental classifications and outcomes. The error rates for each of the ten runs across all eight experiments are within ±1%, demonstrating the high stability and repeatability of our method.

Analyzing Table 5, it can be observed that the improved inception module, the CBAM, and the DropBlock method all improve the effectiveness of DenseNet-I to a certain extent, as applied to the diagnosis of different wear forms in slippers, among which the addition of the attention mechanism has the greatest impact on the enhancement of the categorization ability of the model, and compared with the non-addition of that module, it improves the accuracy rate by 5.1%. The four sets of comparison experiments, 5, 6, 7, and 8, show that the performance of the model cannot be optimized without any of the modules. In Experiment 8, DenseNet-I with all three modules added has the best categorization ability, with accuracy, precision, recall, and F1 scores of 97.3%, 96.5%, 97.5%, and 97.0%, respectively. The results of this experiment show that after adding the improved inception module, CBAM, and DropBlock method, the fault diagnosis performance of the system is significantly increased, and the method has a better result in diagnosis.

### 4.4. Comparative Models

To further validate the advantage of DenseNet-I applied to the fault diagnosis of different wear forms of slippers, it is compared with various classical fault diagnosis network models in the same dataset as the comparison experiments, for example, ResNet18 [35], and 10 experiments are averaged. Table 6 shows the results.

Combining the characteristics of each network model and analyzing the diagnostic outcomes in Table 6, it can be seen that LeNet5 [36], which consists of two convolution layers and three fully connected layers, is a simpler model with the lowest classification accuracy. The classification accuracies of ResNet18 and GoogleNet [37] are 96.1% and 95.8%, respectively, which shows that the inclusion of residual blocks and a multi-channel structure in the network is optimized for the task of fault diagnosis of multiple forms of slipper wear. VGG16 [38] performs relatively well in this experiment with a recognition accuracy of 96.7%, and its use of small- and medium-sized convolutional kernels and deeper network depth enhances the recognition capacity of the network. DenseNet-I has the highest accuracy among the five different fault diagnosis models in the task of fault diagnosis of different forms of slipper wear, with an accuracy of 97.3%, representing that the model has a strong capacity to discriminate between faulty and non-faulty samples in general. Its evaluation metrics, such as the F1 score, are also high compared to other network models. The F1 score is a reconciled average of precision and recall, and an F1 score of 0.973 indicates that the present model strikes a good balance between precision and recall and is both accurate and comprehensive in predicting faults. The high recall of the model indicates that the model is able to capture the faulty samples well with little underreporting. By comparing the fault diagnosis effect of this model with traditional convolutional neural networks, the effectiveness of introducing the improved inception module, CBAM, and the DropBlock method in DenseNet is demonstrated, as well as the superior performance, strong generalization ability, and good fault diagnosis effect of the proposed network, DenseNet-I, in similar fault diagnosis tasks.

**Figure 13:**
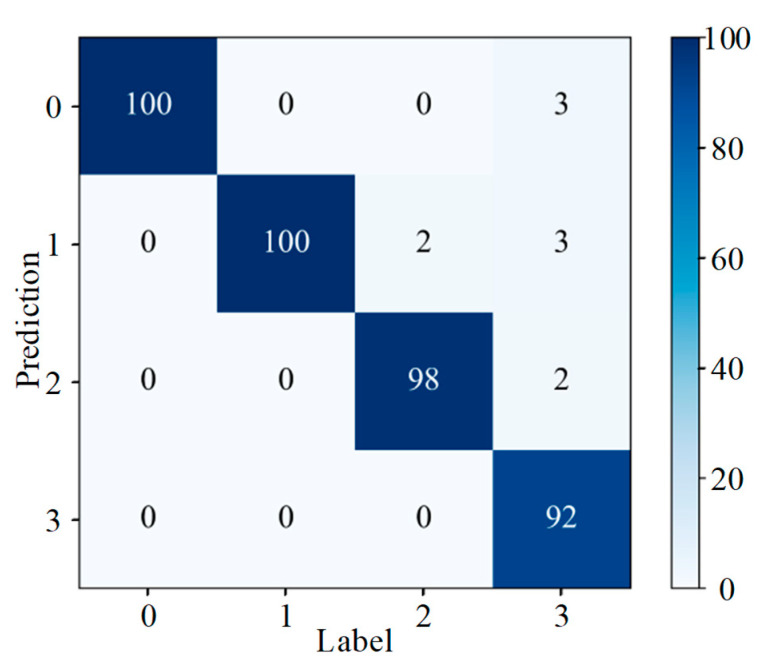
Confusion matrix (different wear forms).

To intuitively evaluate the categorization effectiveness of a model, data mining and analysis are often performed using statistical tools and visualization methods such as a confusion matrix, which provides an intuitive representation of how well a model correctly and incorrectly identifies a particular dataset. Figure 13 represents the confusion matrix generated for the application of the DenseNet-I model to slipper wear fault diagnosis, where the horizontal coordinates are the given labels of the dataset and the vertical coordinates are the predicted labels of the model for a given input data. It can be concluded from the figure that for the 100 samples of corrosive slipper wear faults from label 2, two samples were misidentified as abrasive slipper wear faults from label 1, and for the 100 test samples of adhesive slipper wear from label 3, the number of samples identified as being normal, or showing abrasive or corrosive wear of the slipper were 3, 3, and 2, respectively. The model’s recognition rate for the four faults is at a relatively high level overall.

**Figure 14:**
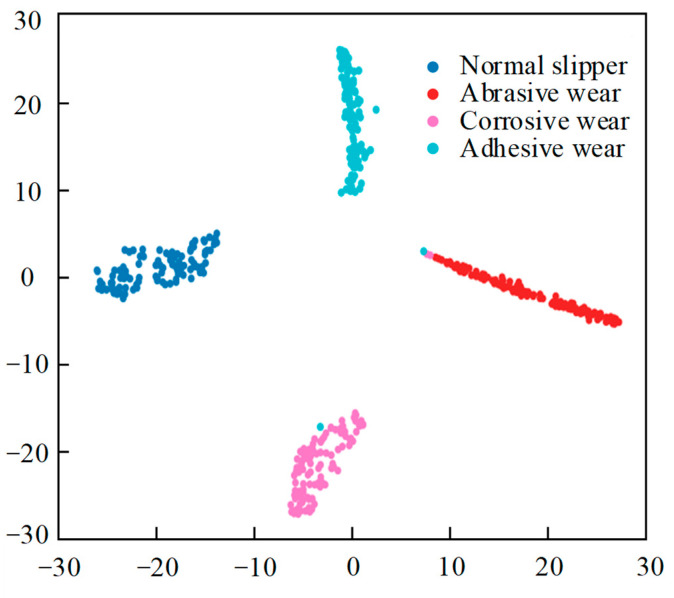
t-SNE feature visualization (different wear forms).

To better observe the feature learning ability of the DenseNet-I fault diagnosis model and examine its performance, this paper adopts the t-SNE dimension reduction visualization method to perform nonlinear dimension reduction operations on the high-dimensional features learned from the fully connected layer, and the generated t-SNE visualization diagram is shown in Figure 14. Each data point on Figure 14 represents a test sample, and the values of the horizontal and vertical axes represent the result of the dimension reduction in this sample point, with different fault types corresponding to points of different colors on the image. By viewing the figure, the feature data points of the four diverse states of the slipper can be clearly and regularly aggregated in different locations, which proves that the DenseNet-I model can achieve a high degree of aggregation of the same kind of data after feature learning and extraction of the input data, and that there is a clear demarcation between the different kinds of data, which represents that the DenseNet-I model built has a powerful feature learning and classification capability. It can be shown that the constructed DenseNet-I model has powerful feature learning and classification abilities, which can produce higher accuracy in recognizing the four forms of piston pump slipper wear.

### 4.5. Fault Diagnosis of Slipper Wear with Different Wear Degrees and Composite Wear Forms

To confirm the generalization of the DenseNet-I model proposed in this article for application to other datasets, the model is used in this section to perform fault diagnosis for different wear degrees of the slipper on individual slippers, as well as for different slipper composite wear forms. Referring to the construction idea and diagnostic process in Section 4.2, the dataset for this two-group experiment is constructed and divided. The first set is used to construct the dataset of different wear degrees of the slipper, as shown in Table 7, with the fault categories of normal, slight, moderate, and severe wear of the slipper. Each category contains 1000 samples. The second group is used to construct the dataset of different composite wear forms of slippers, as shown in Table 8. The composite wear forms are slipper normal, adhesive wear, and corrosive wear slipper failure, adhesive wear and abrasive wear slipper failure, corrosive wear and abrasive wear slipper failure, and adhesive wear and corrosive wear and abrasive wear slipper failure. Each category contains 1000 samples.

**Figure 15:**
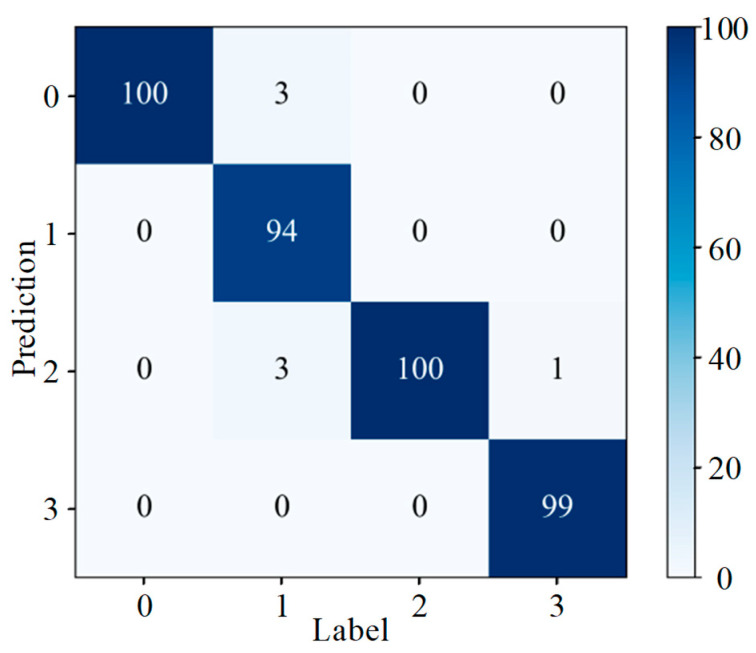
Confusion matrix (different wear degrees).

**Figure 16:**
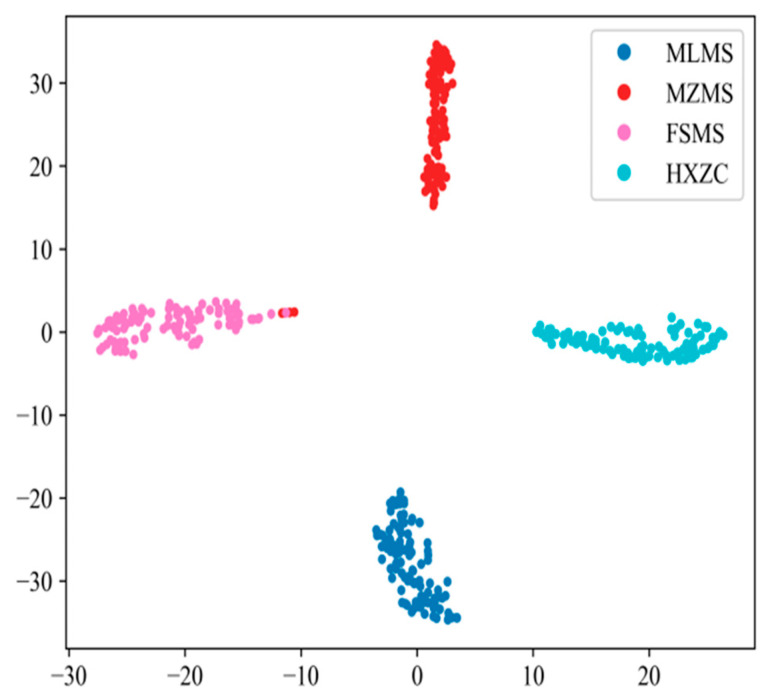
t-SNE feature visualization (different wear degrees).

**Figure 17:**
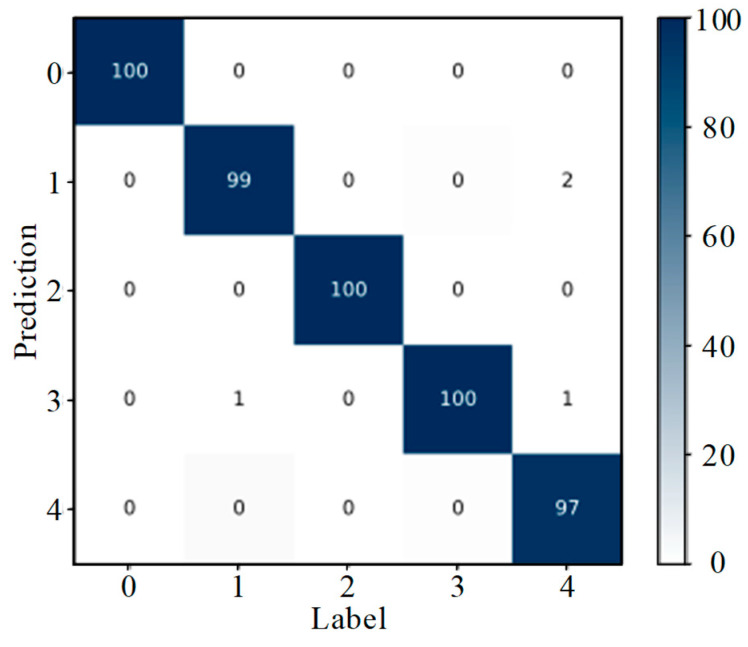
Confusion matrix (different composite wear forms).

**Figure 18:**
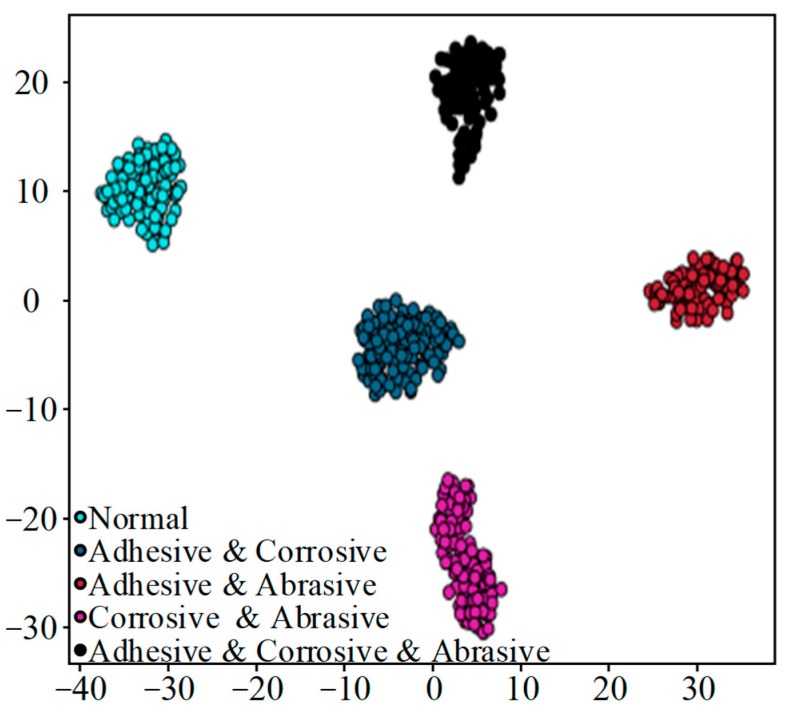
t-SNE feature visualization (different composite wear forms).

The DenseNet-I model hyperparameters and the critical parameters of each module are the same as in Section 4.4. Experiments are conducted on the SDP image dataset with different wear degrees on a single slipper and on the SDP image datasets with different composite wear forms on a slipper. At the end of the experiment, the capacity of the model on the two test datasets is analyzed and studied using the confusion matrix and the t-SNE visualization tool. The outcomes associated with the testing of individual slipper datasets with different wear degrees are plotted as shown in Figure 15 and Figure 16. The results related to the testing of different slipper composite wear form datasets are plotted as shown in Figure 17 and Figure 18.

Analysis of Figure 15 shows that, of the 100 test samples with label 1 (slight wear), three were misidentified as label 0 (slipper normal), and another three were misidentified as label 2 (moderate wear). Only one of the test samples with label 3 (serious wear) was misidentified as label 2 (moderate wear); all other samples were correctly classified and identified. As can be seen from Figure 17, only 1 out of 100 samples comprising label 1 (composite wear forms of slipper with adhesive wear and corrosive wear) was misidentified as label 3 (composite wear forms of slipper with corrosive wear and abrasive wear). Out of 100 samples comprising label 4 (composite wear forms of slipper with adhesive wear and corrosive wear, and abrasive wear), 2 were misidentified as label 1 (composite wear forms of slipper with adhesive wear and corrosive wear), and 1 was misidentified as label 3 (composite wear forms of slipper with corrosive wear and abrasive wear). All other samples were correctly classified and identified.

Figure 16 and Figure 18 tell us that the feature points of the four classes of samples with different degrees of wear on one single slipper and the five classes of samples with different slipper wear forms are highly clustered. The categories are distant from each other with clear boundaries. The above analyses show that DenseNet-I has a good classification performance and feature-learning ability in the problem of diagnosing slipper faults with different wear degrees on single slippers, as well as slipper faults with different slipper composite wear forms. This experiment verifies the generalization capacity of the model when applied to diverse datasets.

To further validate the generalization ability of the proposed framework, additional experiments were conducted using the Case Western Reserve University (CWRU) bearing dataset. The results show that our method achieves a diagnostic accuracy of over 98%, outperforming comparative baseline models, including LeNet-5 and VGG16, which both yielded accuracies below 98% under identical experimental conditions. This demonstrates the strong transferability and robustness of our approach across different mechanical systems.

## 5. Conclusions

In the paper, a fault diagnosis method based on SDP and multi-channel DenseNet is proposed for slipper wear fault diagnosis of swashplate axial piston pumps. The method transforms the triaxial vibration signals into SDP images as inputs to the model and replaces the small convolutional kernel in the inception module with a larger convolution kernel to raise the feature extraction capacity of the network. In addition, the introduction of the CBAM into DenseNet raises the accuracy of fault diagnosis. Replacing the existing ReLU function in the network with a Leaky ReLU function solves the problem of neuron deactivation in networks. A DropBlock regularization method was also added to DenseNet to raise the generalization ability of the network. Sufficient experiments were conducted on several SDP image datasets, and the method performed well. The method is able to solve similar fault diagnosis problems with better accuracy and generalization compared to other state-of-the-art methods. Around algorithmic programming management, we have retained the unique functional effects of DenseNet and made innovative optimizations to strengthen the differentiation of the DenseNet-I algorithm for similar features and simplify the computation so that it can be used in the domain of fault diagnosis of various similar features.

In the process of conducting this study, the slipper wear faults were injected manually, which differs from the actual faults of axial piston pumps; meanwhile, the fault type, load pressure, and rotational speed of the test piece were always stable and unchanged during the experimental process, which is different from the actual conditions of axial piston pumps, and the capacity of the model in complex industrial working conditions has yet to be verified.

In future work, the fault injection method will be optimized to further enhance the availability of the proposed method by taking into account the variations in fault types, fluctuations of load pressure, and changes in rotational speed that exist in the real conditions of the piston pump industry.

## Figures and Tables

**Table 1:** Literature review.

Literature	Problem
[1,2,3,4,5,6]	Piston pump failure study
[11,12,13]	Typical fault diagnosis
[7,14,15,16,17]	Typical diagnosis of piston pumps
[8,9,10]	Typical deep learning algorithms
[18,19,20,21]	Fault diagnosis with similar characteristics

**Table 2:** The structure and parameters of DenseNet-I.

Structural Layer	Feature Output	Network Parameters
Improved inception	256 × 256	Multi-scale convolution
Convolution layer	128 × 128	7 × 7 Conv, Stride 2
Pooling layer	64 × 64	3 × 3 Max Pool, Stride 2
DenseBlock 1	64 × 64	$\left[\begin{array}{l} 1\times 1\mathrm{Conv}\\ 3\times 3\mathrm{Conv} \end{array}\right]\times 6$
CBAM 1	64 × 64	Attention mechanism
Transition Layer 1	64 × 64 32 × 32	1 × 1 Conv 2 × 2 Average Pool, Stride 2
DenseBlock 2	32 × 32	$\left[\begin{array}{l} 1\times 1\mathrm{Conv}\\ 3\times 3\mathrm{Conv} \end{array}\right]\times 12$
CBAM 2	32 × 32	Attention mechanism
Transition Layer 2	64 × 64 32 × 32	1 × 1 Conv 2 × 2 Average Pool, Stride 2
DenseBlock 3	16 × 16	$\left[\begin{array}{l} 1\times 1\mathrm{Conv}\\ 3\times 3\mathrm{Conv} \end{array}\right]\times 24$
CBAM 3	16 × 16	Attention mechanism
Transition Layer 3	16 × 16 8 × 8	1 × 1 Conv 2 × 2 Average Pool, Stride 2
Classification layer	4 × 4	8 × 8 Global Average Pool Fully connected, Softmax
Improved inception	256 × 256	Multi-scale convolution
Convolution layer	128 × 128	7 × 7 Conv, Stride 2
Pooling layer	64 × 64	3 × 3 Max Pool, Stride 2
DenseBlock 1	64 × 64	$\left[\begin{array}{l} 1\times 1\mathrm{Conv}\\ 3\times 3\mathrm{Conv} \end{array}\right]\times 6$

**Table 3:** SDP images of slipper abrasive wear vibration signals under different parameters.

Experiment Parameter	ξ=20°	ξ=40°	ξ=50°	ξ=60°
l=1	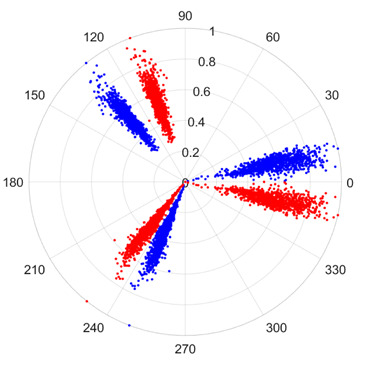	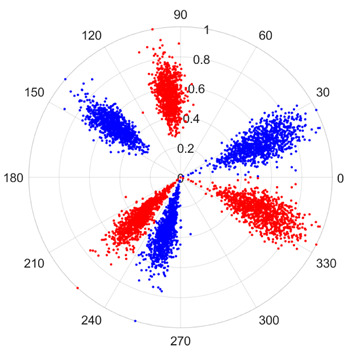	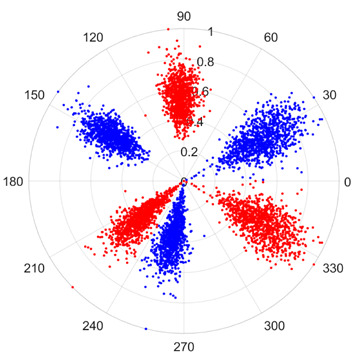	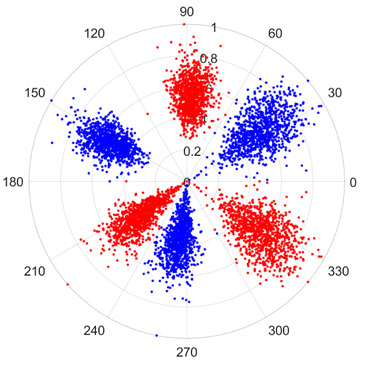
l=3	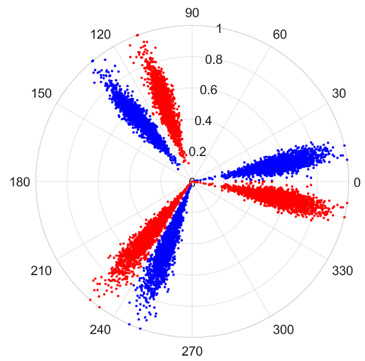	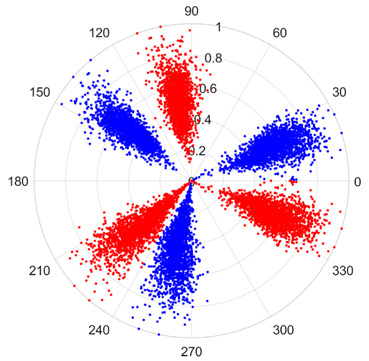	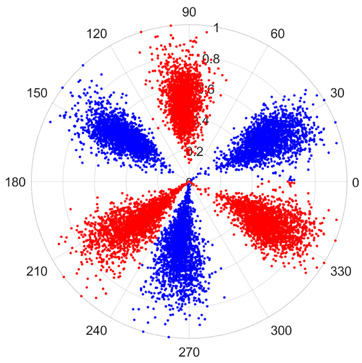	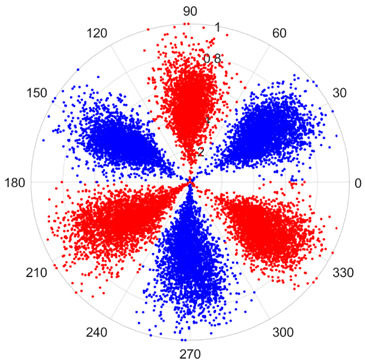
l=5	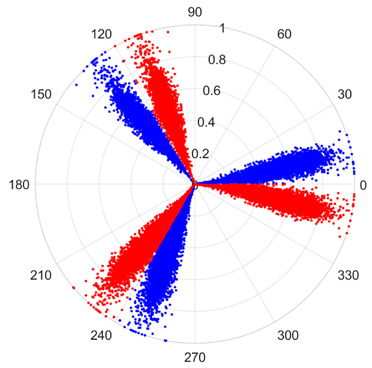	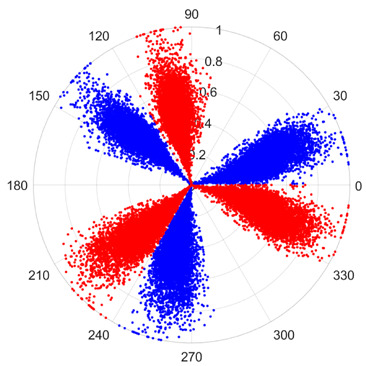	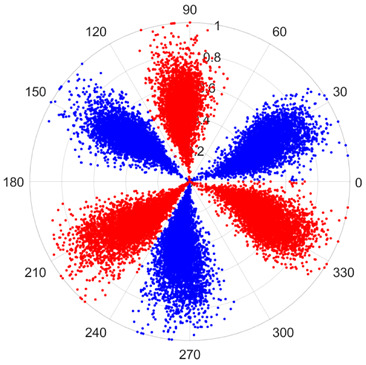	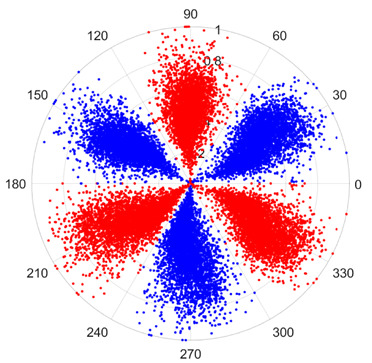
l=7	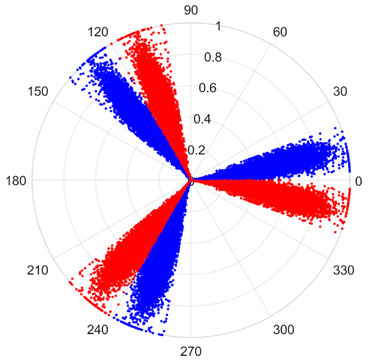	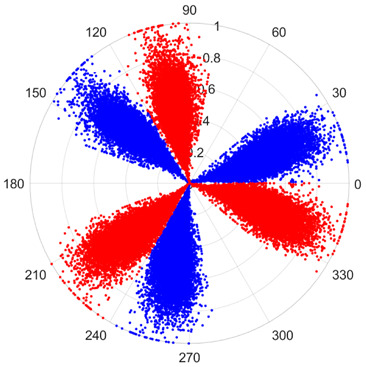	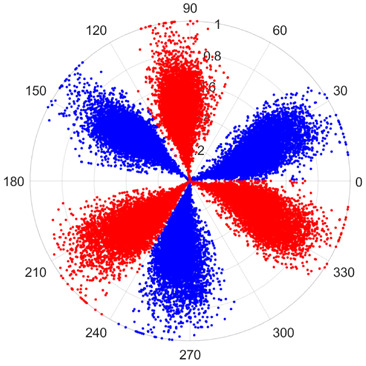	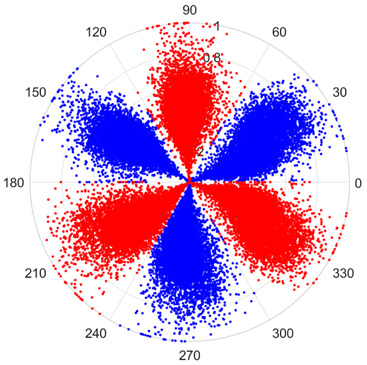
l=10	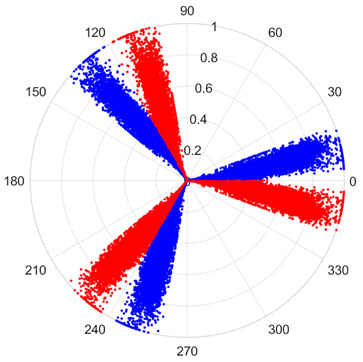	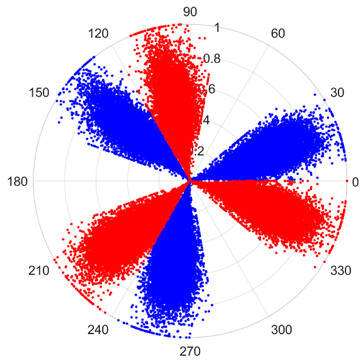	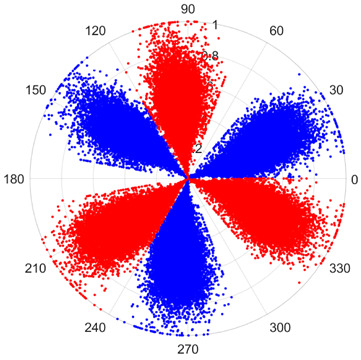	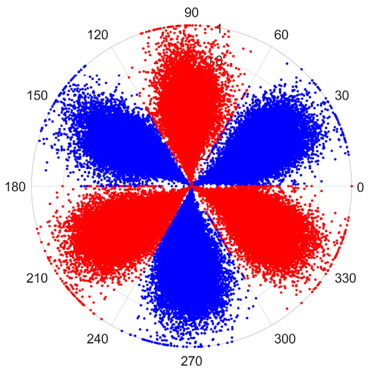

**Table 4:** SDP image dataset of different wear forms.

Slipper Wear Form	Label	Training Samples	Testing Samples	Aggregate
Normal slipper	0	900	100	1000
Abrasive wear	1	900	100	1000
Corrosive wear	2	900	100	1000
Adhesive wear	3	900	100	1000

**Table 5:** Comparison experiment of adding different modules to DenseNet-I.

Experimental Serial Number	Improved Inception Module	CBAM	DropBlock Method	Ac(%)	Pr(%)	Re(%)	F1(%)
1				88.5	86.6	90.4	88.4
2	●			92.3	91.6	94.2	92.3
3		●		93.6	92.1	95.2	93.5
4			●	92.8	91.6	93.9	92.7
5	●	●		96.3	95.6	96.2	95.9
6		●	●	95.6	93.5	97.2	95.2
7	●		●	94.1	93.5	94.9	94.1
8	●	●	●	97.3	96.5	97.5	97.0

In the table, ● represents the addition of this module to DenseNet-I.

**Table 6:** Comparison of experimental results of different network models.

Experimental Serial Number	Name of the Model	Ac(%)	Pr(%)	Re(%)	F1(%)
1	LeNet5	93.2	94.3	92.6	92.8
2	VGG16	96.7	96.1	96.7	96.3
3	GoogleNet	95.8	94.7	97.2	95.8
4	ResNet18	96.1	95.8	96.5	96.0
5	DenseNet-I	97.3	96.7	97.9	97.3

**Table 7:** SDP image dataset of different slipper wear degrees.

Slipper Wear State	Label	Training Samples	Testing Samples	Aggregate
Normal slipper	0	900	100	1000
Slight wear	1	900	100	1000
Moderate wear	2	900	100	1000
Serious wear	3	900	100	1000

**Table 8:** SDP image dataset of different slipper composite wear forms.

Slipper Wear State	Label	Training Samples	Testing Samples	Aggregate
Normal slipper	0	900	100	1000
Adhesive wear and Corrosive wear	1	900	100	1000
adhesive wear and abrasive wear	2	900	100	1000
corrosive wear and abrasive wear	3	900	100	1000
adhesive wear and corrosive wear, and abrasive wear	4	900	100	1000
Slipper wear state	Label	Training samples	Testing samples	Aggregate

## Data Availability

The original contributions presented in this study are included in the article. Further inquiries can be directed to the corresponding author(s).
